# Impfen in der Dermatologie 2025: Update mit Berücksichtigung aktueller Empfehlungen der Ständigen Impfkommission

**DOI:** 10.1111/ddg.15785_g

**Published:** 2025-08-11

**Authors:** Johanna Stoevesandt, Marc Schmalzing, Sophia Mohme, Matthias Goebeler

**Affiliations:** ^1^ Klinik und Poliklinik für Dermatologie Venerologie und Allergologie Universitätsklinikum Würzburg; ^2^ Medizinische Klinik und Poliklinik II Rheumatologie/Klinische Immunologie Universitätsklinikum Würzburg; ^3^ Hautarztpraxis Dr. Mohme, Porta Westfalica

**Keywords:** COVID‐19, Herpes zoster, Hochdosisimpfstoff, Immunsuppression, Influenza, Mpox, Pneumokokken, respiratorisches Synzytial‐Virus, COVID‐19, herpes zoster, high‐dose vaccine, immunosuppression, influenza, Mpox, pneumococci, respiratory syncytial virus

## Abstract

Die immunsuppressive und ‐modulierende Behandlung dermatologischer Patienten macht eine Überprüfung und gegebenenfalls Aktualisierung notwendiger Standard‐ und Indikationsimpfungen erforderlich. Die Ständige Impfkommission (STIKO) am Robert Koch‐Institut veröffentlicht regelmäßig evidenzbasierte Impfempfehlungen, die an die aktuelle epidemiologische Situation und Verfügbarkeit von Impfstoffen angepasst werden. Seit 2020 ergaben sich mehrere Neuerungen, die auch für Patienten mit dermato(onko)logischen Erkrankungen relevant sind: *(1)* Mit COVID‐19 wurde eine neue virale Erkrankung definiert und mehrere Impfstoffe zu deren Prävention eingeführt; *(2)* in Reaktion auf den weltweiten Mpox‐Ausbruch 2022 erhielt ein 2013 zur Prävention der Pocken zugelassener, auf dem modifizierten Vacciniavirus Ankara basierender, nicht vermehrungsfähiger Lebendimpfstoff eine Indikationserweiterung; *(3)* für die Influenza‐Schutzimpfung von Personen ≥ 60 Jahren wurde ein neuer inaktivierter Hochdosisimpfstoff zugelassen; *(4)* für die Pneumokokkenschutzimpfung steht ein neuer 20‐valenter Konjugatimpfstoff zur Verfügung; *(5)* zuletzt neu zugelassen wurden zwei rekombinante Impfstoffe gegen das respiratorische Synzytial‐Virus (RSV). Die vorliegende Arbeit erörtert die entsprechend angepassten STIKO‐Empfehlungen für Erwachsene mit besonderer Würdigung ihrer Umsetzung bei immunkompromittierten Patienten in der Dermatologie.

## EINLEITUNG

Die Verordnung immunsuppressiver und ‐modulierender Medikamente, beispielsweise zur Behandlung der Psoriasis, des atopischen Ekzems oder blasenbildender Autoimmundermatosen und die Behandlung von Patienten mit fortgeschrittenen dermatologischen Tumorleiden ziehen spezielle Impfempfehlungen nach sich. Innerhalb des dermatologischen Fachgebietes werden darüber hinaus impfpräventable Erkrankungen, insbesondere der Herpes zoster und gelegentlich andere Virusexantheme behandelt. Umfassende Impfempfehlungen mit Relevanz in der Dermatologie wurden unter Berücksichtigung des Einflusses häufig verwendeter Immunsuppressiva und Immunmodulatoren von Mohme et al. im *Journal der Deutschen Dermatologischen Gesellschaft* 2020 zusammengestellt.^1^ Seit der Veröffentlichung ergaben sich, nicht zuletzt infolge der COVID‐19‐Pandemie und eines weltweiten Mpox‐Ausbruches sowie der Zulassung neuer Impfstoffe, relevante Änderungen. Die angepassten Empfehlungen der Ständigen Impfkommission (STIKO) am Robert Koch‐Institut für Erwachsene mit besonderer Würdigung der Umsetzung bei immunkompromittierten, dermatologischen Patienten stellen den Schwerpunkt dieses Updates dar.^2^ Tabelle 1 fasst die mit Stand 01/2025 gültigen, angepassten Empfehlungen zusammen. Vollständige Impfempfehlungen für Erwachsene (Tabelle S1, Online‐Supplement) sowie ergänzend eine Übersicht zu den wichtigsten Indikationsimpfungen immunsupprimierter Kinder‐ und Jugendlicher (Tabelle S2, Online‐Supplement) stehen als Online‐Supplement zur Verfügung.

**TABELLE 1:** Impfempfehlungen für immungesunde und immunkompromittierte/chronisch kranke Erwachsene.^*^

Immungesunde Erwachsene	Immunsupprimierte/chronisch kranke Erwachsene	Impfstoffe^**^; Impfschemata; ggf. Anmerkungen
***COVID‐19***		
*Standardimpfung* Erwachsener inklusive Schwangerer ab dem 2. Trimenon bei unvollständiger Basisimmunität; jährliche Impfung von Personen ≥ 60 Jahren; jährliche *Indikationsimpfung* von engen Kontaktpersonen Immundefizienter und Bewohnern von Pflegeinrichtungen; jährliche *berufliche Impfung* von medizinischem/pflegerischem Personal	Jährliche Auffrischimpfung von Personen mit Immunsuppression und/oder anderen Risikofaktoren (z. B. chronische Erkrankung von Atemwegen, Leber, Niere, Herz/Kreislauf oder ZNS, aktives Tumorleiden, Diabetes mellitus, Adipositas, Trisomie 21); bei starker Immunsuppression ggf. zusätzliche Impfungen und serologische Kontrollen	mRNA‐ (Comirnaty^®^, Spikevax^®^) und proteinbasierte (z. B. Nuvaxovid^®^) Impfstoffe mit jeweils der durch die WHO empfohlenen Variantenanpassung; Basisimmunität besteht bei ≥ 3 Antigenkontakten, davon mindestens 1 Impfung; die genannten Indikationsgruppen erhalten eine jährliche Auffrischung im Herbst
***Herpes zoster***		
*Standardimpfung* von Personen ≥ 60 Jahren	Impfung von Personen ≥ 50 Jahren bei angeborener/erworbener Immundefizienz oder chronischer Erkrankung (z. B. HIV‐Infektion, rheumatoide Arthritis, systemischer Lupus erythematodes, chronisch‐entzündliche Darmerkrankung, Erkrankung der Atemwege, Diabetes mellitus); bislang nicht durch STIKO‐Empfehlungen gedeckte Überlegungen betreffen die Impfung von Personen ≥ 18 Jahren unter Einnahme von JAK‐Inhibitoren und eine passive Immunisierung von Patienten mit Herpes zoster und zu erwartendem, schwerem Verlauf mit Varizella‐zoster‐Immunglobulin^20^	Rekombinanter, adjuvantierter Totimpfstoff (Shingrix^®^); 2‐malige Impfung im Abstand von 2 bis maximal 6 Monaten; die Impfung Erwachsener < 50 Jahren erfolgt außerhalb der offiziellen STIKO‐Empfehlungen (die Kostenübernahme ist individuell zu klären)
***Influenza***		
*Standardimpfung* von Personen ≥ 60 Jahren; *Indikationsimpfung* von Schwangeren ab dem 2. (bei erhöhter Gefährdung 1.) Trimenon, Haushaltskontakten immunsupprimierter Personen, Bewohnern von Alten‐ und Pflegeheimen sowie gemäß Empfehlung der Gesundheitsbehörden bei drohender schwerer Epidemie; *berufliche Impfung* von medizinischem Personal und Personen mit umfangreichem Publikumsverkehr oder Kontakt zu Wildvögeln	Impfung bei erhöhter Gefährdung durch eine chronische Erkrankung (z. B. Atemwegs‐, kardiovaskuläre, neurologische oder Nierenerkrankung, Diabetes mellitus) bzw. angeborene oder erworbene Immundefizienz einschließlich immunsuppressiver Therapie	Inaktivierte Impfstoffe (z. B. Influsplit^®^, Influvac^®^) bzw. Hochdosisimpfstoffe (Efluelda^®^) und adjuvantierte Impfstoffe (Fluad^®^); jeweils jährliche Impfung im Herbst mit einem Impfstoff entsprechend der aktuellen, von der WHO empfohlenen Antigenkombination; Personen ≥ 60 Jahren erhalten unabhängig vom jeweiligen Immunstatus den Hochdosisimpfstoff oder adjuvantierten Impfstoff
***Mpox (ehemals „Affenpocken“)***		
Keine Standardimpfung, *Indikationsimpfung* von Männern mit sexuellem Kontakt zu wechselnden männlichen Partnern; *berufliche Impfung* von Laborpersonal mit gezieltem Kontakt zu infektiösen Orthopox‐Viren; *postexpositionelle Impfung* nach engem körperlichen Kontakt oder längerem Face‐to‐Face‐Kontakt zu Erkrankten, medizinisches Personal nach engem Kontakt ohne adäquate Schutzausrüstung, Laborpersonal nach ungeschütztem Kontakt zu infektiösem Material	Immunsupprimierte mit entsprechender Impfindikation erhalten unabhängig vom Pockenimpfstatus 2 Impfdosen und werden bei Lieferengpass priorisiert geimpft	Nicht replikationsfähiger Lebendimpfstoff (Imvanex^®^), basierend auf modifiziertem Vacciniavirus Ankara; 2 subkutane Impfstoffdosen im Abstand von ≥ 28 Tagen; bei Immungesunden mit Z. n. früherer Pockenimpfung ist 1 Impfdosis ausreichend; die *postexpositionelle Impfung* noch asymptomatischer Kontaktpersonen soll spätestens 14 Tage nach dem Mpox‐Kontakt veranlasst werden
***Pneumokokken***		
*Standardimpfung* von Personen ≥ 60 Jahren, *berufliche Impfung* bei Exposition zu Metallrauchen	Impfung erwachsener Personen mit angeborenen oder erworbenen Immundefekten, unter immunsuppressiver Therapie, mit chronischen Erkrankungen (betreffend z. B. Herz und Kreislauf, Atmungsorgane, Stoffwechsel, Malignome) und mit lokalen Risikofaktoren für eine Pneumokokken‐Meningitis (z. B. Cochlea‐Implantat, Liquorfistel)	20‐valenter Konjugatimpfstoff PCV‐20 (Prevenar 20^®^); einmalige Impfung auch bei Z. n. früherer Impfung mit anderen Pneumokokkenimpfstoffen; der Mindestabstand von 6 Jahren zur letzten Gabe von PPSV‐23 kann bei ausgeprägter Immundefizienz auf 1 Jahr verkürzt werden; der Mindestabstand zur letzten Gabe von PCV‐13 beträgt 1 Jahr
***Respiratorisches Synzytial‐Virus (RSV)***		
*Standardimpfung* von Personen ≥ 75 Jahren; *Indikationsimpfung* von Bewohnern von Pflegeeinrichtungen ≥ 60 Jahren	Impfung von Erwachsenen ≥ 60 Jahren ‐ mit schwerer angeborener oder erworbener Immundefizienz ‐ mit schwerer chronischer Erkrankung (z. B. Erkrankung der Atmungsorgane, Herzkreislauf‐ oder Nierenerkrankung, hämatoonkologische Erkrankung, Diabetes mellitus mit Komplikationen)	Zwei rekombinante, proteinbasierte Impfstoffe (monovalent/adjuvantiert: Arexvy^®^; bivalent/nicht‐adjuvantiert: Abrysvo^®^); Impfung als Einmalgabe vor Eintritt in die anstehende RSV‐Saison

* Tabelle 1 beschränkt sich auf die im Text besprochenen Impfungen; siehe Tabelle S1 (Online‐Supplement) für eine vollständige Zusammenfassung aktueller Impfempfehlungen für immungesunde und immunkompromittierte Erwachsene und Tabelle S2 (Online ‐Supplement) zur Umsetzung der besprochenen Indikationsimpfungen bei Kindern und Jugendlichen.

** Eine umfassende Liste verfügbarer Impfstoffe findet sich auf der Internet‐Seite des Paul‐Ehrlich‐Instituts (https://www.pei.de/DE/arzneimittel/impfstoffe/impfstoffe‐node.html).

Durch eine personelle Neubesetzung ergaben sich 2024 Veränderungen auch innerhalb der STIKO selbst. So wurden zusätzlich Experten aus den Bereichen Kommunikation und Modellierung berufen und die maximale Berufungszeit auf drei jeweils dreijährige Perioden beschränkt.

## IMPFEN ALS WEG AUS DER PANDEMIE: NEUE IMPFEMPFEHLUNGEN FÜR NEUE VIREN

### SARS‐CoV‐2/COVID‐19

Die COVID‐19‐Pandemie war Anlass für die Entwicklung, klinische Prüfung und globale Verteilung neuer Impfstoffe in beispielloser Geschwindigkeit.^3^ Die STIKO empfiehlt aktuell ausschließlich die Verwendung mRNA‐ oder proteinbasierter Impfstoffe, die jeweils entsprechend der von der *World Health Organization* (WHO) empfohlenen Variantenanpassung aktualisiert werden. Alle Erwachsenen sollen eine sogenannte Basisimmunität aufweisen.^2^ Diese wird durch drei Antigenkontakte erreicht, wobei mindestens ein Kontakt einer Impfung entsprechen sollte. Eine jährliche Auffrischimpfung im Herbst wird Individuen mit erhöhtem Risiko für einen schweren Krankheitsverlauf und Personen mit einem erhöhten Kontakt‐ bzw. Übertragungsrisiko einschließlich medizinischem Personal angeraten (Tabelle 1). Bei Immunsupprimierten mit besonders starker Einschränkung der Immunantwort (zum Beispiel nach Organtransplantation), können zum Erreichen der Basisimmunität weitere Impfdosen im Mindestabstand von 4 Wochen erforderlich werden;^2^, ^4^, ^5^ der Impferfolg wird hier durch die Bestimmung spezifischer Antikörper gegen das SARS‐CoV‐2‐Spikeprotein serologisch kontrolliert. Wenn trotz wiederholter Impfung keine ausreichende Immunantwort erzielt wird, kommen gegebenenfalls die Verwendung erhöhter Impfstoffdosen im Off‐Label‐Gebrauch oder ein Präparatewechsel in Betracht.^4^ Zur Aufrechterhaltung der Schutzwirkung kann bei schwerer Immunsuppression gegebenenfalls eine mehrmals jährliche Auffrischimpfung sinnvoll sein.^2^ Zusätzliche Impfdosen sind auch bei Therapie mit Rituximab in Betracht zu ziehen.^6^ Wichtiger erscheint aber die Empfehlung, die Impfung idealerweise 4 Wochen vor oder 6 Monate nach Gabe von Rituximab durchzuführen. Die 2022 für die passive Immunisierung beziehungsweise Präexpositionsprophylaxe zugelassene Antikörperkombination Tixagevimab/Cilgavimab (Evusheld^®^) soll gemäß der angepassten STIKO‐Empfehlung vom Februar 2023 aufgrund einer stark reduzierten Neutralisationskapazität gegenüber den zirkulierenden Omikron‐Sublinien nur noch in begründeten Einzelfällen eingesetzt werden. Eine Gabe kann unter B‐Zell‐depletierender Therapie als mögliche additive Präventionsmaßnahme erwogen werden.^7^

### Mpox

Steigende Fallzahlen von Mpox (ehemals Affenpocken) als Manifestation eines weltweiten Ausbruchs von Mpox‐Viren der Klade II wurden 2022 auch in Deutschland registriert.^8^ Hautkliniken und dermatologische Praxen, insbesondere in Großstädten, sind häufig erste Anlaufstelle bei Mpox‐Infektion. Dermatologen sollten die Erkrankung daher bewusst in ihre Differenzialdiagnostik einbeziehen und auch mit entsprechenden Behandlungs‐ und Impfempfehlungen vertraut sein.^9^ Imvanex^®^, ein auf dem modifizierten Vacciniavirus Ankara basierender, im Menschen nicht vermehrungsfähiger Lebendimpfstoff, ist seit 2013 in der *Europäischen Union* zur Prävention der Pocken zugelassen und erhielt 2022 die Indikationserweiterung zum Schutz vor Mpox. Bei fehlender Verfügbarkeit kann der Impfstoff Jynneos^®^ des gleichen Herstellers eingesetzt werden. Die STIKO empfiehlt eine postexpositionelle Mpox‐Impfung ungeimpfter, erwachsener Personen mit sexuellem Kontakt oder längerem ungeschützten Face‐to‐Face‐Kontakt zu Erkrankten (Tabelle 1).^2^, ^10^ Diese Empfehlung gilt auch für medizinisches Personal, wenn eine Exposition ohne ausreichende Schutzausrüstung (mindestens Mundnasenschutz und Schutzkittel) erfolgte. Zusätzlich rät die STIKO zur Indikationsimpfung von Personen mit erhöhtem Expositionsrisiko.^10^ Hierzu zählen erwachsene Männer, die über Sexualkontakte mit häufig wechselnden, männlichen Partnern berichten. Die Grundimmunisierung beinhaltet zwei subkutane Impfdosen im Abstand von mindestens 28 Tagen. Bei Immungesunden, die in der Vergangenheit gegen Pocken geimpft wurden, ist eine Impfung ausreichend. Eine Impfung von Immunsupprimierten und – anders als bei früheren, im Menschen replikationsfähigen Pockenimpfstoffen – auch von Patienten mit atopischem Ekzem wird bei entsprechender Indikation ausdrücklich angeraten.^11^ Immundefiziente erhalten unabhängig von einer früheren Pockenimpfung zwei Impfstoffdosen und sollen im Falle von Lieferengpässen aufgrund des erhöhten Risikos für einen schweren Verlauf mit Priorität geimpft werden.^10^, ^12^

Eine veränderte Situation ergibt sich durch den ostafrikanischen Ausbruch von Mpox‐Viren der im Vergleich zur Klade II virulenteren Klade Ib, welcher im August 2024 zum Ausruf einer gesundheitlichen Notlage von internationaler Tragweite durch die WHO führte und im Zuge dessen vereinzelte Fälle auch außerhalb des afrikanischen Kontinents registriert wurden. Von einer Wirksamkeit der verfügbaren Impfstoffe wird ausgegangen.

## UPDATE ZU DEN WICHTIGSTEN INDIKATIONSIMPFUNGEN FÜR IMMUNSUPPRIMIERTE: INFLUENZA, PNEUMOKOKKEN, HERPES ZOSTER

### Influenza

Die jährliche Impfung im Herbst mit einem inaktivierten, an die von der WHO empfohlene Antigenkombination angepassten Grippeimpfstoff wird für Personen ≥ 60 Jahren sowie für Schwangere, Bewohner von Pflegeeinrichtungen, chronisch Kranke/Immunsupprimierte und deren Kontaktpersonen angeraten (Tabelle 1). Eine berufliche Impfindikation besteht unter anderem für medizinisches Personal. Seit 2021 empfiehlt die STIKO für Personen ≥ 60 Jahren einen inaktivierten Hochdosisimpfstoff,^13^ welcher mit 60 µg anstelle von 15 µg im Vergleich zu konventionellen Präparaten eine vierfache Antigenmenge enthält. Entsprechend einer STIKO‐Empfehlung vom Oktober 2024 kann für die genannte Altersgruppe alternativ ein MF‐59‐adjuvantierter Impfstoff eingesetzt werden. Die altersspezifische Impfempfehlung wird durch eine bei Senioren erhöhte Wahrscheinlichkeit für Hospitalisierung und schwere Verläufe bei gleichzeitig, unter anderem im Rahmen der Immunseneszenz verminderter Immunantwort auf die Grippeimpfung begründet.^14^ Durch Verwendung des Hochdosisimpfstoffes beziehungsweise adjuvantierten Impfstoffes werden laborbestätigte Influenzainfektionen gegenüber dem konventionellen Präparat geringfügig, aber signifikant reduziert; die Reaktogenität bezüglich impfbedingter Lokal‐ und leichter Allgemeinreaktionen gilt als etwas erhöht.^13^ Übereinstimmend mit den Empfehlungen der WHO rät die STIKO seit August 2024 zum Einsatz tri‐ statt tetravalenter Influenza‐Impfstoffe unter Verzicht auf die B/Yamagata‐Linie, deren Anteil bei der weltweiten Zirkulation stark abgenommen hat. Die Verfügbarkeit trivalenter, inaktivierter Influenza‐Impfstoffe wird ab der Saison 2025/2026 erwartet.

### Pneumokokken

Immungesunden Personen ≥ 60 Jahren, erwachsenen Berufstätigen mit Exposition zu Metallrauchen und chronisch Kranken/Immunsupprimierten wird eine einmalige Impfung mit dem 20‐valenten Konjugatimpfstoff (PCV‐20) Prevenar 20^®^ angeraten (Tabelle 1). Die Empfehlung zur Gabe von PCV‐20 löste 2023 die Vorgaben zur Standardimpfung mit dem 23‐valenten Polysaccharidimpfstoff (PPSV‐23) beziehungsweise zur seriellen Impfung Immunsupprimierter mit dem 13‐valenten Konjugatimpfstoff (PCV‐13) und PPSV‐23 ab.^15^ Sie wird durch eine gegenüber PPSV‐23 überlegene Immunogenität und gegenüber PCV‐13 breitere Serotypenabdeckung begründet; vergleichende Daten zur klinischen Wirksamkeit existieren nicht.^15^, ^16^ PCV‐20 kann gleichzeitig mit einem Influenzaimpfstoff oder mit einem RNA‐basierten COVID‐19‐Impfstoff verabreicht werden;^17^ zur Notwendigkeit von Wiederholungsimpfungen liegen keine Daten vor. Prevenar 20^®^ erhielt im März 2024 eine Zulassungserweiterung für Kinder ≥ 6 Wochen; die STIKO empfiehlt bislang jedoch weiterhin die Verwendung von PCV‐13/PCV‐15 (Grundimmunisierung ab 2 Monaten) beziehungsweise zusätzlich von PPSV‐23 (chronisch Kranke zwischen 2 und 17 Jahren).

### Herpes zoster

Die Zoster‐Schutzimpfung wird unverändert allen Personen ≥ 60 Jahren und chronisch Kranken/Immunsupprimierten bereits ab 50 Jahren angeraten (Tabelle 1). Aktuelle Daten weisen auf einen protektiven Effekt der Vakzine gegenüber Demenzerkrankungen hin.^18^ Über die gültigen STIKO‐Empfehlungen hinausgehend rät die *Deutsche Gesellschaft für Rheumatologie und klinische Immunologie* (DGRh) erwachsenen Patienten, die einen Januskinase(JAK)‐Inhibitor (zum Beispiel Baricitinib, Tofacitinib, Upadacitinib) einnehmen, zur Impfung bereits ab einem Alter von 18 Jahren (https://dgrh.de/Start/Versorgung/Therapieinformationen/Therapieinformationsbögen.html). Diese Empfehlung begründet sich durch das – abhängig vom verwendeten JAK‐Inhibitor unterschiedlich stark erhöhte – Risiko einer Zostervirus‐Reaktivierung.^19^ Sie ist auf die Einnahme von JAK‐Inhibitoren bei dermatologischer Indikation übertragbar. Zu beachten ist, dass die Impfung Erwachsener < 50 Jahren in der Schutzimpfungsrichtlinie des *Gemeinsamen Bundesausschusses* (https://www.g‐ba.de/richtlinien/60/) nicht aufgeführt und damit trotz Zulassung nicht regelhaft erstattungsfähig ist.

Aktuelle, ebenfalls über die STIKO‐Empfehlungen hinausgehende Überlegungen betreffen eine passive Immunisierung von Personen, welche akut an Herpes zoster erkrankt sind und bei denen aufgrund von Vorerkrankungen ein schwerer Verlauf beziehungsweise eine Generalisierung zu befürchten ist. Einige historische Arbeiten sowie eine kleine aktuelle Fallserie implizieren,^20^ dass die Gabe von Varizella‐zoster‐Immunglobulin zusätzlich zur antiviralen Standardtherapie den Krankheitsverlauf günstig beeinflussen kann. Eine Beobachtungsstudie (EU PAS Nummer: EUPAS103611), in die 120 mit Varizella‐zoster‐Immunglobulin behandelte Patienten und 40 Kontrollen eingeschlossen werden sollen, wurde im Juni 2023 durch den Hersteller initiiert.

## NEUE IMPFOPTION FÜR EINEN ALTBEKANNTEN ERREGER: RESPIRATORISCHES SYNZYTIAL‐VIRUS

Das weltweit verbreitete respiratorische Synzytial‐Virus (RSV) verursacht Erkrankungen der Atemwege in jedem Lebensalter. Schwere und mitunter lebensbedrohliche Verläufe betreffen zumeist Säuglinge und Kleinkinder, insbesondere bei Koinfektion mit Bakterien, Pilzen oder anderen Viren, jedoch auch ältere, vorerkrankte und immunsupprimierte Personen.^21^ Mit den rekombinant hergestellten Impfstoffen Arexvy^®^ (monovalent, adjuvantiert) und Abrysvo^®^ (bivalent) stehen seit 2023 zwei Präparate für die Schutzimpfung zur Verfügung. Der adjuvantierte Impfstoff ist ausschließlich für Personen ≥ 50 Jahren zugelassen, Abrysvo^®^ zusätzlich für die Immunisierung Schwangerer, um deren Neugeborenen/Säuglingen einen passiven Schutz zu vermitteln. Die STIKO rät seit August 2024 zur einmaligen Standardimpfung von Personen ≥ 75 Jahren. Patienten mit angeborener oder erworbener Immundefizienz beziehungsweise schwerer chronischer Erkrankung (betreffend zum Beispiel Atmungsorgane, Nieren, Herz‐Kreislaufsystem, blutbildendes System) sowie Bewohnern von Pflegeeinrichtungen wird bereits ab 60 Jahren eine RSV‐Impfung angeraten (Tabelle 1).^22^ Mit Palivizumab und seit 2022 zusätzlich Nirsevimab stehen zwei monoklonale, gegen das virale F‐Protein gerichtete Antikörper für die RSV‐Prophylaxe bei Säuglingen und Kleinkindern zur Verfügung. Die STIKO rät mit Beschluss vom Juni 2024 zur einmaligen Passiv‐Immunisierung von Neugeborenen/Säuglingen vor Eintritt in deren erste RSV‐Saison mit Nirsevimab.^23^

## UMGANG MIT IMPFSKEPTIKERN

Durch die rasante Entwicklung neuer Impfstoffe und die globale Impfkampagne im Rahmen der COVID‐19‐Pandemie wurde das Thema „Impfen“ zum Gegenstand persönlicher und politischer Auseinandersetzungen zwischen Impfbefürwortern, ‐gegnern und zahlreichen Unentschlossenen.^24^ Auch Patienten mit dermatologischen Erkrankungen und/oder geplanter immunsuppressiver Therapie stehen notwendigen Impfungen nicht selten mit Skepsis gegenüber.^25^ Für das aufklärende Gespräch stellt das RKI „Faktensandwiches“ zur Verfügung, die häufige Impfmythen und Fehlinformationen gezielt aufgreifen und durch sachliche Informationen erklärend einordnen (https://www.rki.de/DE/Themen/Infektionskrankheiten/Impfen/Informationsmaterialien/Impfmythen/falschinformationen‐wirksam‐aufklaeren‐node.html).

## FAZIT

Die immunsuppressive beziehungsweise ‐modulierende Behandlung dermatologischer Patienten macht eine regelmäßige Überprüfung und Aktualisierung notwendiger Standard‐ und Indikationsimpfungen erforderlich.^1^ Hautärzte sollten mit den gültigen STIKO‐Empfehlungen (Tabelle 1) vertraut und sich bewusst sein, dass im Falle einer veränderten epidemiologischen Situation und/oder der Entwicklung neuer Impfstoffe regelmäßig Anpassungen vorgenommen werden, über die das *Epidemiologische Bulletin* als Publikationsorgan des RKI einmal jährlich zusammenfassend informiert.^2^

## DANKSAGUNG

Open access Veröffentlichung ermöglicht und organisiert durch Projekt DEAL.

## INTERESSENKONFLIKT

Keiner.

## Supporting information



Supporting information
