# Supplementary material for: Impfen in der Dermatologie 2025: Update mit Berücksichtigung aktueller Empfehlungen der Ständigen Impfkommission
Source: J Dtsch Dermatol Ges. 2025 Aug 11;23(8):925–31. [Article in German] doi: 10.1111/ddg.15785_g (PMC12338436; doi:10.1111/ddg.15785_g)
Supplement: Supplementary file 1 — Supporting information [file DDG-23-925-s001.docx]

Ergänzende Materialien (Tabellen S1 und S2)

Impfen in der Dermatologie 2025: Update mit Berücksichtigung aktueller Empfehlungen der Ständigen Impfkommission

Vaccination in dermatology 2025: update considering current recommendations of the German Standing Committee on Vaccination

Johanna Stoevesandt^1^, Marc Schmalzing^2^, Sophia Mohme^1,3^, Matthias Goebeler^1^

^1^Klinik und Poliklinik für Dermatologie, Venerologie und Allergologie,
Universitätsklinikum Würzburg, Würzburg

^2^Medizinische Klinik und Poliklinik II, Rheumatologie/Klinische Immunologie, Universitätsklinikum Würzburg, Würzburg

^3^Hautarztpraxis Dr. Mohme, Porta Westfalica

## **Tabelle S1** Impfempfehlungen für immungesunde und immunkompromittierte/chronisch kranke Erwachsene.

**Table S1** Vaccination recommendations for immunocompetent and immunocompromised/chronically ill adults.

| **Immungesunde Erwachsene** | **Immunsupprimierte/chronisch kranke Erwachsene** | **Impfstoffe; Impfschemata; ggf. Anmerkungen** |
| --- | --- | --- |
| COVID-19 |  |  |
| **Standardimpfung Erwachsener inklusive Schwangerer ab dem 2. Trimenon bei unvollständiger Basisimmunität; jährliche Impfung von Personen ≥ 60 Jahren;**  **jährliche Indikationsimpfung von engen Kontaktpersonen Immundefizienter und Bewohner von Pflegeinrichtungen;**  **jährliche berufliche Impfung von medizinischem/pflegerischem Personal** | **Jährliche Auffrischimpfung von Personen mit Immunsuppression und/oder anderen Risikofaktoren (z.B. chronische Erkrankung von Atemwegen, Leber, Niere, Herz/Kreislauf oder ZNS, aktives Tumorleiden, Diabetes mellitus, Adipositas, Trisomie 21); bei starker Immunsuppression ggf. zusätzliche Impfungen und serologische Kontrollen** | **mRNA- (Comirnaty®, Spikevax®) und proteinbasierte (z.B. Nuvaxovid®) Impfstoffe mit jeweils der durch die WHO empfohlenen Variantenanpassung; Basisimmunität besteht bei ≥ 3 Antigenkontakten, davon mindestens 1 Impfung; die genannten Indikationsgruppen erhalten eine jährliche Auffrischung im Herbst** |
| Diphtherie |  |  |
| Standardimpfung bzw. Nachholimpfung von Personen mit fehlender oder unvollständiger Grundimmunisierung; Auffrischimpfung alle 10 Jahre; postexpositionelle Impfung nach engem Face-to-Face Kontakt zu Erkrankten, wenn die letzte Impfung > 5 Jahre zurückliegt | Entsprechend Empfehlung für Immungesunde | Diphtherie-Toxoid; Grundimmunisierung durch 3 Impfdosen; Erwachsene erhalten die nächste fällige Auffrischimpfung einmalig als Tdap- bzw. ggf. Tdap-IPV Kombination |
| Frühsommer-Meningoenzephalitis (FSME) |  |  |
| Keine Standardimpfung; Indikationsimpfung zeckenexponierter Personen in Risikogebieten; berufliche Impfung von exponiertem Laborpersonal und von Forst-/Landwirten in Risikogebieten | Entsprechend Empfehlung für Immungesunde | Inaktiviertes FSME-Virus; Grundimmunisierung und regelmäßige Auffrischimpfung gemäß Angaben in den produktspezifischen Fachinformationen; **Liste der FSME-Risikogebiete wird regelmäßig aktualisiert** |
| Haemophilus influenzae B |  |  |
| Keine Standardimpfung Erwachsener | Impfung bei anatomischer oder funktioneller Asplenie (z.B. Sichelzellkrankheit) | Konjugatimpfstoff; einmalige Impfung der Indikationsgruppen; Bezug monovalenter Impfstoffe über Auslandsapotheke |
| Hepatitis A |  |  |
| Keine Standardimpfung, Indikationsimpfung von Personen mit erhöhter sexueller Exposition, Konsumierenden intravenöser Drogen, von Bewohnern psychiatrischer Einrichtungen; berufliche Impfung von Tätigen im Gesundheitsdienst, in Kindertagesstätten, Wohnheimen, bei Kontakt zu Abwasser; Reiseimpfung vor Aufenthalt in Endemiegebieten; postexpositionelle Impfung v.a. in Gemeinschaftseinrichtungen | Impfung von Patienten mit chronischen Erkrankungen, die eine häufige Übertragung von Blutbestandteilen erforderlich machen (z.B. Hämophilie) oder mit Krankheiten der Leber | Inaktiviertes Hepatitis A-Virus, monovalente Impfstoffe und Kombinationen mit rekombinantem Hepatitis B-Impfstoff; Grundimmunisierung und ggf. Auffrischimpfung gemäß Angaben in den produktspezifischen Fachinformationen; postexpositionelle Impfung mit monovalentem Impfstoff innerhalb von 14 Tagen nach Exposition |
| Hepatitis B |  |  |
| Keine Standardimpfung Erwachsener;  Indikationsimpfung bei erhöhter Exposition (z.B. Haushaltskontakte von Hepatitis B-Antigenträgern, Strafgefangene, Bewohner psychiatrischer Einrichtungen); berufliche Impfung z.B. von medizinischem Personal, Polizisten, Personal in Einrichtungen mit erhöhter Prävalenz von Hepatitis B-Infizierten; Reiseimpfung nach individueller Gefährdungsbeurteilung; postexpositionelle Impfung unzureichend immuner Personen nach Kanülen-Stichverletzung/Blutkontakt zu infektiösem Material (ggf. simultan mit Hepatitis B-Immunglobulin) | Impfung von Patienten, bei denen aufgrund einer vorbestehenden oder zu erwartenden Immundefizienz, immunsuppressiver Therapie oder chronischer Erkrankung (z.B. HIV, Hepatitis C, dialysepflichtige Niereninsuffizienz) mit einem schweren Verlauf gerechnet werden muss; die Impfindikation ist auf Grundlage des tatsächlichen Expositionsrisikos zu stellen | Verschiedene rekombinante Impfstoffe, Grundimmunisierung gemäß Angaben in den produktspezifischen Fachinformationen; bereits als Kinder Geimpfte werden bei besonderer Exposition ein weiteres Mal geimpft; 4-8 Wochen nach Abschluss der Impfserie sind serologische Kontrollen und für „Low-/Non-Responder“ zusätzliche Impfungen indiziert; serologische Kontrollen und ggf. Auffrischungen für erfolgreich Geimpfte werden bei humoraler Immundefizienz (1x/Jahr) oder beruflich/privat verstärkter Exposition (erstmals 10 Jahre nach Impfung) angeraten |
| Herpes zoster |  |  |
| Standardimpfung von Personen ≥ 60 Jahren | Impfung von Personen ≥ 50 Jahren bei angeborener/erworbener Immundefizienz oder chronischer Erkrankung (z.B. HIV-Infektion, rheumatoide Arthritis, systemischer Lupus erythematodes, chronisch-entzündliche Darmerkrankung, Erkrankung der Atemwege, Diabetes mellitus); **bislang nicht durch STIKO-Empfehlungen gedeckte Überlegungen betreffen die Impfung von Personen ≥ 18 Jahren unter Einnahme von JAK-Inhibitoren und eine passive Immunisierung von Patienten mit Herpes zoster und zu erwartendem, schwerem Verlauf mit Varizella-zoster-Immunglobulin** | Rekombinanter, adjuvantierter Totimpfstoff (Shingrix®); 2-malige Impfung im Abstand von 2 bis maximal 6 Monaten; die Impfung Erwachsener < 50 Jahren erfolgt außerhalb der offiziellen STIKO-Empfehlungen (die Kostenübernahme ist individuell zu klären) |
| Humanes Papillomavirus |  |  |
| Keine Standardimpfung Erwachsener; Erwachsene ≥ 18 Jahren können von einer Impfung profitieren , wobei die Wirksamkeit bei nicht HPV-naiven Personen reduziert ist | Ggf. Impfung von Erwachsenen mit HIV-Infektion oder systemischem Lupus erythematodes | Rekombinante Impfstoffe; Grundimmunisierung gemäß Angaben in den produktspezifischen Fachinformationen; die Impfung Erwachsener erfolgt außerhalb der offiziellen STIKO-Empfehlungen (die Kostenübernahme ist individuell zu klären) |
| Influenza |  |  |
| Standardimpfung von Personen ≥ 60 Jahren; Indikationsimpfung von Schwangeren ab dem 2. (bei erhöhter Gefährdung 1.) Trimenon, Haushaltskontakten immunsupprimierter Personen, Bewohnern von Alten- und Pflegeheimen sowie gemäß Empfehlung der Gesundheitsbehörden bei drohender schwerer Epidemie; berufliche Impfung von medizinischem Personal und Personen mit umfangreichem Publikumsverkehr oder Kontakt zu Wildvögeln | Impfung bei erhöhter Gefährdung durch eine chronische Erkrankung (z.B. Atemwegs-, kardiovaskuläre, neurologische oder Nierenerkrankung, Diabetes mellitus) bzw. angeborene oder erworbene Immundefizienz einschließlich immunsuppressiver Therapie | Inaktivierte Impfstoffe (z.B. Influsplit®, Influvac®) bzw. **Hochdosisimpfstoffe (Efluelda®) und adjuvantierte Impfstoffe (Fluad®)**; jeweils jährliche Impfung im Herbst mit einem Impfstoff entsprechend der aktuellen, von der WHO empfohlenen Antigenkombination; **Personen ≥ 60 Jahren erhalten unabhängig vom jeweiligen Immunstatus den Hochdosisimpfstoff oder adjuvantierten Impfstoff** |
| Masern |  |  |
| Standardimpfung nach 1970 geborener Erwachsener mit unklarem Impfstatus, ohne Impfung oder mit nur einer Impfung; Indikationsimpfung (seit 01.03.2020 Pflichtimpfung gemäß §20 IfSG) bei bevorstehender Aufnahme bzw. beim Besuch einer Gemeinschaftseinrichtung (z.B. Kindergarten, Schule); im Rahmen von Ausbrüchen; berufliche Impfung nach 1970 geborener Personen mit Tätigkeit in Gesundheitseinrichtungen wie Krankenhäusern und Arztpraxen oder in Gemeinschaftseinrichtungen/-unterkünften (seit 01.03.2020 Pflichtimpfung) sowie bei Tätigkeit mit Kontakt zu potenziell infektiösem Material; postexpositionelle Impfung nicht oder nicht sicher immuner Kontaktpersonen | Lebendimpfungen sind unter laufender Immunsuppression (abhängig von Wirkstoff und Dosis) kontraindiziert; empfängliche Immundefiziente und Schwangere erhalten nach Kontakt zu Masernkranken eine Postexpositionsprophylaxe mit Standardimmunglobulinen (Cave: Erfolg einer späteren MMR-Impfung ist in den 8 Monaten nach Immunglobulingabe eingeschränkt) | Verschiedene attenuierte Lebendimpfstoffe, ausnahmslos Kombinationsimpfstoffe (MMR, MMR-V); die Standardimpfung Erwachsener sowie die Impfung im Rahmen von Ausbrüchen beinhalten eine MMR-Impfdosis; bei den anderen genannten Impfindikationen sind 2 dokumentierte Dosen erforderlich |
| Meningokokken |  |  |
| Keine Standardimpfung Erwachsener; berufliche Impfung von exponiertem Laborpersonal; Indikationsimpfung bei Ausbrüchen gemäß Empfehlung der Gesundheitsbehörden; Reiseimpfung bei Aufenthalt in Ländern mit epidemischem Vorkommen; postexpositionelle Impfung ungeimpfter Haushalts- oder vergleichbar enger Kontakte | Impfung (Serogruppen ACWY+B) von Patienten mit angeborener oder erworbener Immundefizienz, insbesondere bei  - Komplement-/Properdindefizienz  - Therapie mit C5-Komplementinhibitoren  - Hypogammaglobulinämie  - anatomischer oder funktioneller Asplenie (z.B. bei Sichelzellanämie) | Konjugatimpfstoffe gegen Serogruppen A, C, W und Y; rekombinant hergestellte Impfstoffe auf Proteinbasis gegen Serogruppe B; Grundimmunisierung gemäß Angaben in den produktspezifischen Fachinformationen |
| Mpox (ehemals „Affenpocken“) |  |  |
| **Keine Standardimpfung, Indikationsimpfung von Männern mit sexuellem Kontakt zu wechselnden männlichen Partnern; berufliche Impfung von Laborpersonal mit gezieltem Kontakt zu infektiösen Orthopox-Viren; postexpositionelle Impfung nach engem körperlichen Kontakt oder längerem Face-to-face Kontakt zu Erkrankten, medizinisches Personal nach engem Kontakt ohne adäquate Schutzausrüstung, Laborpersonal nach ungeschütztem Kontakt zu infektiösem Material** | **Immunsupprimierte mit entsprechender Impfindikation erhalten unabhängig vom Pockenimpfstatus 2 Impfdosen und werden bei Lieferengpass priorisiert geimpft** | **Nicht replikationsfähiger Lebendimpfstoff (Imvanex®), basierend auf modifiziertem Vacciniavirus Ankara; 2 subkutane Impfstoffdosen im Abstand von ≥ 28 Tagen; bei Immungesunden mit Z. n. früherer Pockenimpfung ist 1 Impfdosis ausreichend; die postexpositionelle Impfung noch asymptomatischer Kontaktpersonen soll spätestens 14 Tage nach dem Mpox-Kontakt veranlasst werden** |
| Mumps |  |  |
| Berufliche Impfung nach 1970 geborener Personen mit Tätigkeit in Gesundheitseinrichtungen wie Krankenhäusern und Arztpraxen oder in Gemeinschaftseinrichtungen/-unterkünften sowie bei Tätigkeit mit Kontakt zu potenziell infektiösem Material; postexpositionelle Impfung von nicht oder nicht sicher immunen Kontaktpersonen | Lebendimpfungen sind unter laufender Immunsuppression (abhängig von Wirkstoff und Dosis) kontraindiziert | Verschiedene attenuierte Lebendimpfstoffe, ausnahmslos Kombinationsimpfstoffe (MMR, MMR-V); insgesamt zweimalige Impfung ist erforderlich |
| Pertussis |  |  |
| Einmalige Standard- bzw. Auffrischimpfung im Erwachsenenalter; Indikationsimpfung Schwangerer zu Beginn des 3.Trimenons und von Personen mit engem Kontakt zu Neugeborenen (alle 10 Jahre); berufliche Impfung von medizinischem Personal und Personen mit Tätigkeit in Gemeinschaftseinrichtungen (alle 10 Jahre) | Entsprechend Empfehlung für Immungesunde | Azellulärer Komponenten-Adsorbatimpfstoff; monovalente Pertussisimpfstoffe sind nicht erhältlich, die einmalige Auffrischung im Erwachsenenalter erfolgt anlässlich der nächsten fälligen Impfung gegen Tetanus/Diphtherie einmalig als Tdap-Kombination bzw. bei entsprechender Indikation als Tdap-IPV |
| Pneumokokken |  |  |
| Standardimpfung von Personen ≥ 60 Jahren, berufliche Impfung bei Exposition zu Metallrauchen | Impfung erwachsener Personen mit angeborenen oder erworbenen Immundefekten, unter immunsuppressiver Therapie, mit chronischen Erkrankungen (betreffend z.B. Herz und Kreislauf, Atmungsorgane, Stoffwechsel, Malignome) und mit lokalen Risikofaktoren für eine Pneumokokken-Meningitis (z.B. Cochlea-Implantat, Liquorfistel) | **20-valenter Konjugatimpfstoff PCV-20 (Prevenar 20®);** **einmalige Impfung auch bei Z. n. früherer Impfung mit anderen Pneumokokkenimpfstoffen;** **der Mindestabstand von 6 Jahren zur letzten Gabe von PPSV-23 kann bei ausgeprägter Immundefizienz auf 1 Jahr verkürzt werden; der Mindestabstand zur letzten Gabe von PCV-13 beträgt 1 Jahr** |
| Poliomyelitis |  |  |
| Standard- bzw. Auffrischimpfung im Jugend- oder Erwachsenenalter (eine nicht erfolgte Grundimmunisierung soll nachgeholt werden); Indikationsimpfung für Geflüchtete/Asylsuchende aus Gebieten mit Infektionsrisiko; Reiseimpfung bei Aufenthalt in entsprechenden Gebieten; berufliche Impfung von medizinischem Personal mit potentiellem Kontakt zu Erkrankten, Personal in Einrichtungen für Geflüchtete/Asylsuchende, Laborpersonal mit möglichem Infektionsrisiko; postexpositionelle Impfung aller Kontaktpersonen unabhängig von ihrem Impfstatus | Entsprechend Empfehlung für Immungesunde | Inaktivierte Poliomyelitisviren (IPV); zur Verfügung stehen monovalente und Kombinationsimpfstoffe; als vollständig geimpft gelten Personen, die eine Grundimmunisierung (3 Impfdosen) und zusätzlich eine einmalige Auffrischimpfung erhalten haben; die berufliche Impfung wird bei fortbestehender Exposition alle 10 Jahre aufgefrischt |
| Respiratorisches Synzytialvirus (RSV) |  |  |
| **Standardimpfung von Personen ≥ 75 Jahren; Indikationsimpfung von Bewohnern von Pflegeeinrichtungen ≥ 60 Jahren** | **Impfung von Erwachsenen ≥ 60 Jahren**  **- mit schwerer angeborener oder erworbener Immundefizienz**  **- mit schwerer chronischer Erkrankung (z.B. Erkrankung der Atmungsorgane, Herzkreislauf- oder Nierenerkrankung, hämatoonkologische Erkrankung, Diabetes mellitus mit Komplikationen)** | **Zwei rekombinante, proteinbasierte Impfstoffe (monovalent/adjuvantiert: Arexvy®; bivalent/nicht-adjuvantiert: Abrysvo®); Impfung als Einmalgabe vor Eintritt in die anstehende RSV-Saison** |
| Röteln |  |  |
| Berufliche Impfung analog zur Impfung gegen Mumps; Indikationsimpfung für Frauen im gebärfähigen Alter mit unklarem Impfstatus, ohne oder mit nur einer dokumentierten Impfung | Lebendimpfungen sind unter laufender Immunsuppression (abhängig von Wirkstoff und Dosis) kontraindiziert | Verschiedene attenuierte Lebendimpfstoffe, ausnahmslos Kombinationsimpfstoffe (MMR, MMR-V); insgesamt zweimalige Impfung ist erforderlich |
| Tetanus |  |  |
| Standardimpfung bzw. Nachholimpfung von Personen mit fehlender oder unvollständiger Grundimmunisierung; Auffrischimpfung alle 10 Jahre; postexpositionelle Impfung im Verletzungsfall | Entsprechend Empfehlung für Immungesunde | Tetanus-Toxoid; siehe Impfempfehlungen zur Diphtherie; postexpositionelle Impfung ggf. in Kombination mit Tetanus-Immunglobulin (abhängig vom Impfstatus und Art der Verletzung) |
| Varizellen |  |  |
| Indikationsimpfung seronegativer Frauen mit Kinderwunsch, empfänglicher Personen (d.h. negative Erkrankungsanamnese oder seronegativ) mit schwerem atopischen Ekzem und empfänglicher Kontaktpersonen von Risikopersonen (z.B. Schwangere, Immundefiziente, Neugeborene); berufliche Impfung seronegativer Personen bei Tätigkeit in Gesundheitseinrichtungen wie Krankenhäusern und Arztpraxen oder in Gemeinschaftseinrichtungen/-unterkünften sowie bei Tätigkeit mit Kontakt zu potenziell infektiösem Material; postexpositionelle Impfung empfänglicher Personen, die ihrerseits in Kontakt zu Risikopersonen stehen | Lebendimpfungen sind unter laufender Immunsuppression (abhängig von Wirkstoff und Dosis) kontraindiziert; seronegative Personen sollen nach Möglichkeit vor geplanter immunsuppressiver Therapie oder Organtransplantation geimpft werden; die Postexpositionsprophylaxe empfänglicher immundefizienter Personen und Schwangerer erfolgt durch passive Immunisierung mit Varizella-zoster-Immunglobulin, ggf. in Kombination mit antiviraler Chemoprophylaxe | Attenuierte Lebendimpfstoffe; erhältlich sind Monokomponentenimpfstoffe und Kombinationen mit MMR (MMR-V); insgesamt zweimalige Impfung ist erforderlich |

Neuerungen seit 2020 sind fett dargestellt. Standard-Impfempfehlungen für Kinder und Jugendliche sowie ausschließliche Reiseimpfungen und/oder Indikationsimpfungen für Laborpersonal sind nicht berücksichtigt. Eine jeweils umfassende Liste verfügbarer Impfstoffe findet sich auf der Internet-Seite des Paul-Ehrlich-Instituts (https://www.pei.de/DE/arzneimittel/impfstoffe/impfstoffe-node.html)

Changes since 2020 are printed bold. Standard vaccination recommendations for children/adolescents and exclusive travel vaccinations and/or exclusive vaccinations for laboratory personnel are not included. A comprehensive list of available vaccines can be found on the website of the Paul-Ehrlich-Institut (https://www.pei.de/DE/arzneimittel/impfstoffe/impfstoffe-node.html)

Abkürzungen:

FSME Frühsommer-Meningoenzephalitis

IfSG Infektionsschutzgesetz

IPV Inaktivierte Poliomyelitisviren

JAK-Inhibitor Januskinase-Inhibitor

MMR Masern, Mumps, Röteln

MMR-V Masern, Mumps, Röteln, Varizellen

PCV-13 13-valenter Pneumokokken-Konjugatimpfstoff

PCV-20 20-valenter Pneumokokken-Konjugatimpfstoff

PPSV-23 23-valenter Pneumokokken-Polysaccharidimpfstoff

STIKO Ständige Impfkommission

Tdap Kombinationsimpfstoff aus Tetanus-Toxoid, Diphtherie-Toxoid und azellulärem Komponenten-Adsorbat-Pertussis-Impfstoff

WHO World Health Organization

## **Tabelle S2** Umsetzung der im Text besprochenen Indikationsimpfungen bei Kindern und Jugendlichen

**Table S2** Implementation of the indication vaccinations discussed in the text in children and adolescents

| **Immungesunde Kinder und Jugendliche** | **Immunsupprimierte/chronisch kranke Kinder und Jugendliche** | **Impfstoffe; Impfschemata; ggf. Anmerkungen** |
| --- | --- | --- |
| COVID-19 |  |  |
| Keine Standardimpfung von Kindern/Jugendlichen | Personen ≥ 6 Monaten mit erhöhtem Risiko für einen schweren Erkrankungsverlauf  infolge einer Grunderkrankung bzw. angeborener oder erworbener Immunsuppression | mRNA- (Comirnaty®, Spikevax®) und proteinbasierte (z.B. Nuvaxovid®) Impfstoffe in der durch die WHO empfohlenen Variantenanpassung und mit jeweils altersspezifischer Zulassung/Dosierung; Basisimmunität besteht bei ≥ 3 Antigenkontakten, davon mindestens 1 Impfung; die genannten Indikationsgruppen erhalten eine jährliche Auffrischung im Herbst |
| Herpes zoster |  |  |
| Keine Standardimpfung von Kindern und Jugendlichen | Keine Impfempfehlung für immunsupprimierte Kinder und Jugendliche |  |
| Influenza |  |  |
| Keine Standardimpfung von Kindern und Jugendlichen | Impfung von Personen ≥ 6 Monaten bei erhöhter Gefährdung durch eine chronische Erkrankung bzw. angeborene oder erworbene Immundefizienz einschließlich immunsuppressiver Therapie | Inaktivierte Impfstoffe (z.B. Influsplit®, Influvac®); jeweils jährliche Impfung im Herbst mit einem Impfstoff entsprechend der aktuellen, von der WHO empfohlenen Antigenkombination;  Anmerkung: Im Alter von 2-17 Jahren steht alternativ eine attenuierte Influenza-Lebendvakzine (Fluenz®) zur nasalen Anwendung zur Verfügung, welche bei klinisch manifester Immunschwäche jedoch kontraindiziert ist |
| Pneumokokken |  |  |
| Standardimpfung bzw. Grundimmunisierung von Säuglingen ≥ 2 Monaten (Frühgeborene ebenfalls ab chronologischem Alter ≥ 2 Monaten) | Indikationsimpfung von Kindern und Jugendlichen (2-17 Jahre) mit angeborenen oder erworbenen Immundefekten, chronischen Krankheiten bzw. mit anatomischen und/oder Fremdkörper-assoziierten Risikofaktoren (z.B. Cochlea-Implantat, Liquorfistel) | 13- und 15-valenter Konjugatimpfstoff PCV-13 (Prevenar 13®) bzw. PCV-15 (Vaxneuvance®); 23-valenter Pneumokokken-Polysaccharidimpfstoff PPSV-23 (Pneumovax® 23);  Grundimmunisierung reifgeborener Säuglinge im Alter von 2, 4 und 11 Monaten (2+1);  Grundimmunisierung Frühgeborener im Alter von 2, 3, 4 und 11 Monaten (3+1);  sequentielle Indikationsimpfung mit PCV-13 oder PCV-15, gefolgt von PPSV-23 im Abstand von 6-12 Monaten;  Anmerkung: PCV-20 (Prevenar 20®) ist seit 03/2024 für die Anwendung ab einem Alter von 6 Wochen zugelassen; die STIKO empfiehlt aufgrund der geringeren Immunogenität von PCV-20 vorläufig weiter die Grundimmunisierung mit PCV-13/PCV-15; eine Empfehlung zur Impfung immunsupprimierter Kinder mit PCV-20 liegt ebenfalls nicht vor (Stand 01/2025) |
| Respiratorisches Synzytialvirus (RSV) |  |  |
| Einmalige passive Immunisierung von Neugeborenen und Säuglingen | Entsprechend Empfehlung für Immungesunde | Gegen das virale F-Protein gerichteter, monoklonaler Antikörper Nirsevimab (Beyfortus®);  Säuglinge, die zwischen April und September geboren werden, erhalten Nirsevimab im Herbst vor Eintritt in die RSV-Saison; Neugeborene mit Geburt während der laufenden RSV-Saison (Oktober bis März), erhalten Nirsevimab möglichst rasch (3.–10. Lebenstag) |

Abkürzungen:

PCV-13 13-valenter Pneumokokken-Konjugatimpfstoff

PCV-15 15-valenter Pneumokokken-Konjugatimpfstoff

PCV-20 20-valenter Pneumokokken-Konjugatimpfstoff

PPSV-23 23-valenter Pneumokokken-Polysaccharidimpfstoff

STIKO Ständige Impfkommission
